# Cytosolic and Nucleosolic Calcium Signaling in Response to Osmotic and Salt Stresses Are Independent of Each Other in Roots of *Arabidopsis* Seedlings

**DOI:** 10.3389/fpls.2017.01648

**Published:** 2017-09-21

**Authors:** Feifei Huang, Jin Luo, Tingting Ning, Wenhan Cao, Xi Jin, Heping Zhao, Yingdian Wang, Shengcheng Han

**Affiliations:** Beijing Key Laboratory of Gene Resource and Molecular Development, College of Life Sciences, Beijing Normal University Beijing, China

**Keywords:** *Arabidopsis*, calcium dynamics, cytosolic calcium, nucleosolic calcium, parvabumin, subcellular localization

## Abstract

Calcium acts as a universal second messenger in both developmental processes and responses to environmental stresses. Previous research has shown that a number of stimuli can induce [Ca^2+^] increases in both the cytoplasm and nucleus in plants. However, the relationship between cytosolic and nucleosolic calcium signaling remains obscure. Here, we generated transgenic plants containing a fusion protein, comprising rat parvalbumin (PV) with either a nuclear export sequence (PV-NES) or a nuclear localization sequence (NLS-PV), to selectively buffer the cytosolic or nucleosolic calcium. Firstly, we found that the osmotic stress-induced cytosolic [Ca^2+^] increase (OICI_cyt_) and the salt stress-induced cytosolic [Ca^2+^] increase (SICI_cyt_) were impaired in the *PV-NES* lines compared with the *Arabidopsis* wildtype (WT). Similarly, the osmotic stress-induced nucleosolic [Ca^2+^] increase (OICI_nuc_) and salt stress-induced nucleosolic [Ca^2+^] increase (SICI_nuc_) were also disrupted in the *NLS-PV* lines. These results indicate that PV can effectively buffer the increase of [Ca^2+^] in response to various stimuli in *Arabidopsis*. However, the OICI_cyt_ and SICI_cyt_ in the *NLS-PV* plants were similar to those in the WT, and the OICI_nuc_ and SICI_nuc_ in the *PV-NES* plants were also same as those in the WT, suggesting that the cytosolic and nucleosolic calcium dynamics are mutually independent. Furthermore, we found that osmotic stress- and salt stress-inhibited root growth was reduced dramatically in the *PV-NES* and *NLS-PV* lines, while the osmotic stress-induced increase of the lateral root primordia was higher in the *PV-NES* plants than either the WT or *NLS-PV* plants. In addition, several stress-responsive genes, namely *CML37*, *DREB2A*, *MYB2*, *RD29A*, and *RD29B*, displayed diverse expression patterns in response to osmotic and salt stress in the *PV-NES* and *NLS-PV* lines when compared with the WT. Together, these results imply that the cytosolic and nucleosolic calcium signaling coexist to play the pivotal roles in the growth and development of plants and their responses to environment stresses.

## Introduction

Calcium is commonly accepted as a ubiquitous second messenger in eukaryotic organisms in which it regulates diverse biological processes, such as fertilization, pollen tube elongation, proliferation, neural signaling, and learning (Berridge et al., 2000; Bootman et al., 2001; Cullen and Lockyer, 2002). The cytosolic Ca^2+^ ([Ca^2+^]_cyt_) increases or oscillations arise from an external Ca^2+^ influx, which is primarily mediated by the plasma membrane Ca^2+^ channels, such as CNGCs (DeFalco et al., 2016), GLRs (Davenport, 2002), annexins (Laohavisit et al., 2009) and hyperosmolarity-gated calcium-permeable channel (OSCAs) (Yuan et al., 2014), and/or internal store Ca^2+^ release, which is mediated by endomembrane-localized Ca^2+^ channels, such as the vacuolar Ca^2+^-activated two-pore channel 1 (TPC1) (Peiter et al., 2005). Although the voltage-dependent Ca^2+^ channels and ligand-gated Ca^2+^ channels, such as inositol 1,4,5-trisphosphate- and cyclic ADP ribose-activated channels (Muir and Sanders, 1997; Navazio et al., 2001), have been well characterized via electrophysiological approaches, no molecular identity has been found for these channels in plants to date. In addition, the efflux of [Ca^2+^]_cyt_ is achieved by Ca^2+^-ATPases and/or Ca^2+^/H^+^ anti-porter systems (Kudla et al., 2010), which are responsible for the restoration of [Ca^2+^]_cyt_ to pre-stimulus levels. Therefore, a given Ca^2+^ signal is generated by balancing, in a strictly regulated way, the activation of Ca^2+^ channels, the subsequent inactivation of channels, and the activation of efflux transporters to meet wide-ranging needs in plant growth and development.

Stimulus-specific Ca^2+^ signals, when viewed in terms of the spatial and temporal dynamics of the stimulus-induced changes in [Ca^2+^]_cyt_, have been called the ‘Ca^2+^ signature’ which is characterized by the duration, frequency, amplitude, and spatial location of [Ca^2+^] (McAinsh and Pittman, 2009). The Ca^2+^ signature in plants has two basic patterns: the first is the circadian Ca^2+^ oscillation that occurs at the whole-tissue level (Johnson et al., 1995), which is mainly regulated by the CAS-IP_3_ pathway in *Arabidopsis* (Tang et al., 2007) and is measured by an aequorin-based calcium indicator (Knight et al., 1991); the second is those short-term Ca^2+^ increases or spikes which respond to various abiotic and biotic stimuli, namely light, high and low temperatures, touch, salt and drought, osmotic stress, plant hormones, fungal elicitors, and nodulation factors (Sanders et al., 1999), which can be measured by aequorin and fluorescence resonance energy transfer (FRET)-based yellow cameleon indicators (Miyawaki et al., 1997). In addition, the [Ca^2+^]_cyt_ signals can form a signaling cassette with the reactive oxygen species (ROS) in order to facilitate long-distance systemic responses in each plant, which may provide the feed-forward mechanisms to amplify and transmit the stimuli signals (Choi et al., 2016).

Ca^2+^ is a core regulator in many cellular signal-transduction cascades that modulates gene transcription, which happens in the cell nucleus. It is a dual-membrane organelle bound by the inner and outer nuclear membranes and fused at the nuclear pores. The contiguous perinuclear space within the lumen of the endoplasmic reticulum plays the role of Ca^2+^ storage in signal transduction. So the interesting question is whether nucleosolic Ca^2+^ ([Ca^2+^]_nuc_) is as important as [Ca^2+^]_cyt_ to be involved in gene expression. Analyzing gene transcription in the hippocampal neurons, Hardingham et al. (1997) demonstrated that [Ca^2+^]_nuc_ activates gene transcription by a mechanism distinct from gene regulation as driven by [Ca^2+^]_cyt_. Previous study showed that EGF triggered the [Ca^2+^] increases in both the nucleus and cytosol. However, EGF-induced transactivation of the ternary complex factor, Elk-1, required [Ca^2+^]_nuc_ but not [Ca^2+^]_cyt_ in HepG2 or HEK293 cells (Pusl et al., 2002). Therefore, [Ca^2+^]_nuc_ signaling is generated autonomously or just caused by the passive diffusion from cytoplasm has raised tremendous attention. Mazars and colleagues clearly showed that the delay between [Ca^2+^]_cyt_ peak and [Ca^2+^]_nuc_ in tobacco suspension cells could range from seconds in response to mastoparan (Pauly et al., 2000) to minutes in response to osmotic shocks (Pauly et al., 2001), elicitors (Lecourieux et al., 2005) and sphingolipids (Xiong et al., 2008; Lachaud et al., 2010). In the legume symbiosis signaling pathway, both the rhizobial bacteria nodulation factor and the arbuscular mycorrhizal fungi Myc factor can induce [Ca^2+^]_nuc_ oscillations (Sieberer et al., 2009; Genre et al., 2013), which are sensed by a nuclear-localized CCaMK that activates the different transcriptional regulators required for nodulation and mycorrhization, respectively (Charpentier and Oldroyd, 2013). Previous research has shown that the potassium-permeable channel, DMI1, and the SERCA MCA8 are localized to the nuclear membranes, and they are essential for the nucleosolic Ca^2+^ spiking that occurs in *Medicago truncatula* (Capoen et al., 2011). Furthermore, Charpentier et al. (2016) showed that the three cyclic nucleotide–gated ion channels, CNGC15a/b/c, are located at the nuclear envelope where they form a complex with DMI1, which mediates nuclear Ca^2+^ release and subsequent symbiotic responses in *M. truncatula*. These results highlight the potential of the nucleus to independently generate the Ca^2+^ signals in both plant and animal cells (Bootman et al., 2009; Mazars et al., 2011).

Using tagged versions of Cameleon YC 3.6 (Nagai et al., 2004) that were separately targeted to the cytoplasm, with a nuclear export sequence (NES-YC), and to the nucleoplasm, with a nuclear localization sequence (NLS-YC), Krebs et al. (2012) found that external ATP induced the [Ca^2+^]_nuc_ and [Ca^2+^]_cyt_ increases in the *Arabidopsis* root cells and the Nod factor induced the [Ca^2+^]_nuc_ and [Ca^2+^]_cyt_ spiking in *Lotus japonicus* root hair cells. These results suggested that stimuli could simultaneously induce the Ca^2+^ signaling in the cytosol and nucleus. However, the relationship between cytosolic Ca^2+^ signaling and nucleosolic Ca^2+^ signaling in plants is still not clear. PV is a Ca^2+^-binding protein with three EF hand motifs, one of them is inactive owing to a two amino-acid deletion in the loop region (Cates et al., 2002), and functions as calcium buffer in fast-contracting muscles, brain and some endocrine tissues (Berchtold et al., 1984; Cowan et al., 1990). Prior studies showed that the expression of PV, when selectively targeted to the nucleus (PV-NLS) or cytoplasm (PV-NES), is able to locally attenuate [Ca^2+^]_nuc_ and [Ca^2+^]_cyt_ by 50%, respectively, in response to stimulation with ATP in HepG2 cells (Pusl et al., 2002) or vasopressin in SKHep1 cells (Rodrigues et al., 2007). In the present study, we first generated transgenic *Arabidopsis* plants that selectively expressed PV targeted to the cytoplasm (PV-NES) or nucleus (NLS-PV). Then we crossed the *PV-NES* and *NLS-PV* lines with those of *NES-YC* and *NLS-YC*, respectively, to yield four double-transgenic lines, *NES-YC/PV-NES*, *NLS-YC/NLS-PV*, *NES-YC/NLS-PV*, and *NLS-YC/PV-NES*, for measuring [Ca^2+^]_cyt_ and [Ca^2+^]_nuc_ in the plant response to hyperosmolarity and salt stresses. Using this toolkit, we deciphered the temporal and spatial characteristics of Ca^2+^ signatures in the nucleus and the cytosol in *Arabidopsis* root cells.

## Materials and Methods

### Plant Material and Growth Conditions

*Arabidopsis thaliana* (Col-0 ecotype) plants were grown under 16 h light (120 μmol m^-2^ s^-1^)/8 h dark at 22°C and 60% relative humidity. Seeds of *Arabidopsis* were surface-sterilized by 75% ethanol and plated on Murashige and Skoog (MS) salts, 1% sucrose, 0.8% (w/v) agar, pH 5.8. After stratification at 4°C in the dark for 3 days, the dishes were transferred to a growth chamber for germination and seedling growth of 7 days. About 15–20 seedlings were planted into pots for continued growth and monitored during the experiment. Transgenic plants were generated using the floral-dip method (Clough and Bent, 1998) and screened using the MS medium containing 50 μg/mL Hygromycin. To detect whether the PV buffers the calcium increase in cytoplasm or nucleus, we generated the *Arabidopsis* plants to co-express PV and the calcium indicator Cameleon YC3.6 (YC) by separately crossing three transgenic plants which had nuclear-localized PV (*NLS-PV*) or cytosolic-localized PV (*NES-PV*) with either *NES-YC* or *NLS-YC* transgenic *Arabidopsis* (Krebs et al., 2012), the latter kindly gifted to us from Dr. Jörg Kudla. For each construct, the independent transgenic lines were selected, and the three lines were used for the following experiments.

### DNA Constructs

The CDS of the *PV* gene was cloned from rat muscle tissue. The NLS segment was cloned from the *Arabidopsis BRI1-EMS-SUPPRESSOR 1* (BES1, AT1G19350) gene (Yin et al., 2005) and the NES from the human MKK gene (Tolwinski et al., 1999). The NLS was fused to the N-terminal of PV via PCR, digested by *BamH*I and *Not*I, and cloned into pE2c and pE6c plasmids (Addgene, Cambridge, MA, United States). The NES was fused to the C-terminal of PV, digested by *BamH*I and *Not*I, and cloned into pE2n and pE6n. The primers used to clone these gene fragments are listed in Supplementary Table S1.

The pE2n, pE2c, pE6n, and pE6c constructs containing different PV fragments were used in combination with the destination vector pMDC32^1^ with the Gateway LR II kit (Invitrogen, Carlsbad, CA, United States) to generate all of the plant expression vectors. These were introduced into the *Agrobacterium tumefaciens* strain, GV3101, for the *Arabidopsis* transformation. Construct maps containing both *PV-NES* and *NLS-PV* are shown in **Supplementary Figure S1**. All vectors were confirmed by sequencing.

### Subcellular Localization of NLS-PV and PV-NES in the *Arabidopsis* Mesophyll Protoplasts

The vectors containing either *eYFP-PV-NES* or *NLS-PV-eYFP* and the nuclear marker gene were co-transformed and transiently expressed in the *Arabidopsis* mesophyll protoplasts, as described before (Liang et al., 2015). After incubation for 16 h at 22°C in the dark, fluorescence was visualized by using a Zeiss LSM 700 confocal microscope (Zeiss, Oberkochen, Germany). Observations were made using a 63× objective under oil immersion. The eYFP fluorescence was excited at 488 nm and collected at shortpass 550 infrared (IR). The chloroplast autofluorescence was also excited at 488 nm, but it was collected at longpass 640 IR. The mCherry fluorescence was excited at 555 nm and collected at shortpass 630 IR. The pinhole was approximately 1.0 unit and the thickness of optical section was approximately 0.5 μm. The nuclear marker labeled by mCherry served as the control.

### Protein Extraction and Western Blot Analyses

Four-week-old transgenic *Arabidopsis* leaves were frozen in liquid nitrogen and ground using a mortar and pestle. The samples were incubated on ice for 2 h in an extraction buffer I that contained 50 mM Tris-HCl, pH 8.0, 50 mM NaCl, 5 mM MgCl_2_, 0.1% Triton X-100, 5 mM DTT, and the appropriate protease inhibitor cocktail (Roche, Basel Switzerland), and then centrifuged at 80 000 *g*, at 4°C, for 30 min. The ensuing supernatants were centrifuged again under the same conditions and collected as the cytosolic compartment. The pellet was re-suspended with an extraction buffer II that contained 50 mM Tris-HCl, pH 8.0, 150 mM NaCl, 10% glycerol, 0.1% Triton X-100, 0.1% NP-40, 2 mM MgCl_2_, 5 mM DTT, and 1x protease inhibitor cocktail, and centrifuged again under same conditions as above. The nuclear proteins are in the pellet and were re-suspended with the buffer II in equal volume. In addition, 6-day-old *Arabidopsis* seedlings were collected, ground in liquid nitrogen, and used for the total protein extraction with a buffer (50 mM Tris-HCl, pH 6.8, 2% SDS, 10% glycerin, and 1% β-mercaptoethanol).

Protein extracts were resolved on 12%-SDS-polyacrylamide gels and electro-blotted onto Bio-Rad Immun-Blot PVDF membranes (Bio-Rad, Hercules, CA, United States). After this transfer, the PVDF membranes were blocked in Tris-buffered saline-Tween 20 (TBST; containing 10 mM Tris–HCl, pH 8.0, 150 mM NaCl, and 0.05% Tween 20), which contained 5% bovine serum albumin (Sigma–Aldrich, St. Louis, MO, United States), for 1 h at room temperature and then incubated overnight with a primary antibody at 4°C. The membranes were washed of 10 min for three times in the TBST and incubated with the secondary antibody—anti-rabbit immunoglobulin G, dilution 1:2000 or anti-mouse immunoglobulin G, dilution 1:3000—for 30 min. Next, the membranes were washed of 10 min for three times in the TBST. To examine the alkaline phosphatase activity, the Chemiluminescent Substrate (Roche) was used according to the manufacturer’s protocol. The primary anti-PV antibody (Abcam, dilution 1:2000) was used to detect the expression of PV in the transgenic plants. The non-specific band after blotted with anti-PV antibody was used as the loading control of the cytosolic proteins and the anti-histone antibody (Sigma–Aldrich, dilution 1:10000) was used as the loading control of the nuclear proteins.

### *Arabidopsis* Seedling Preparation for Ca^2+^ Imaging

After germination, the *Arabidopsis* seedlings were grown vertically on the half-strength MS medium for 5–7 days and their roots were prepared for Ca^2+^ imaging following Krebs et al. (2012), with some modifications. The roots were immobilized by overlaying them with 1% (w/v) low-melting-point agarose (Amresco) in the Attofluor^®^Cell Chambers (Invitrogen). After digging a small tunnel in the agarose to expose the root, we gently applied 200 μl bathing solution buffer [0.5x MS salt, 1% sucrose, 10 mM 2-(*N*-morpholino)ethanesulfonic acid [MES]-KOH, pH 5.8] to the chamber. The shoot was not submerged in the solution. High concentrations of NaCl and sorbitol in the same solution were separately perfused as the stimulus into the chamber. The mature zone of the *Arabidopsis* roots was selected for the subsequent Ca^2+^ measurements.

### FRET Measurements

To examine the FRET signals of the transgenic plants, we used an inverted fluorescence microscope (Axio Observer A1; Zeiss) equipped with an iXon3 EMCCD camera (Oxford Instruments, Abingdon, United Kingdom), a Lambda DG4 fluorescent light source (Sutter Instruments, Novato, CA, United States), and Bright Line filter sets (Semrock Inc., Rochester, NY, United States). Images captured with the CFP (438Ex/483Em), CpVenus (500Ex/542Em), and FRET filters (FCFP, FCpVenus and Fraw (438Ex/542Em), respectively, were collected every 3 s at room temperature by using a 40× oil objective (N.A.1.30; Zeiss) and processed in Slidebook v.5.0 software (Intelligent Imaging Innovations, Denver, CO, United States).

The FRET signal was calculated using a previously described formula (Ma et al., 2015): FRETc = Fraw-Fd/Dd ^∗^ FCFP–Fa/Da ^∗^ FCpVenus, where FRETc represents the corrected energy transfer, Fd/Dd represents the measured bleed-through of CFP through the FRET filter (0.826), and Fa/Da is the measured bleed-through of CpVenus through the FRET filter (0.048). To reduce the variation caused by different expression levels in the transgenic plants, the FRETc values were normalized against donor fluorescence (FCFP) to generate an N-FRET (i.e., normalized FRET) signal.

To eliminate the influences of instrument-dependent factors, the apparent FRET efficiency, or Eapp, was calculated using the following equation: Eapp = N-FRET/(N-FRET ± G) (Zal and Gascoigne, 2004), where G (4.59) is the system-dependent factor. It is obtained through a partial CpVenus photo-bleaching method: G = (FRETc - FRETcpost)/(FCFPpost - FCFP), where FRETcpost and FCFPpost are the corresponding FRETc and FCFP values after the partial photo-bleach of CpVenus. The intensity of the light used to bleach CpVenus was carefully chosen so that would not also bleach CFP. All of the fluorescence images were collected and briefly processed in MetaFluor software (Molecular Devices, Sunnyvale, CA, United States); the data were further analyzed with Matlab R2014a and plotted by using GraphPad Prism v.5.0 software. The average FRET measurements in response to the different stimuli represent the value of 20–30 root cells from at least nine independent seedlings, each of which included 3 to 6 root cells. Analysis of statistical significance was performed with the unpaired Student’s *t*-test in the GraphPad Prism 5.0 software. The results are presented as means ± SD.

### Abiotic Treatment, Total RNA Isolation, and Quantitative Real-Time (qRT)-PCR Analysis

*Arabidopsis* seeds of the wildtype (WT), NES-PV, and NLS-PV lines were sowed onto a sterile plate containing 1/2 MS salts, 1% sucrose, 0.8% (w/v) agar, and pH 5.8, and stratified at 4°C in the dark for 3 days. Then, the dishes were transferred to the growth chamber for the germination over 6 days. The seedlings were transferred onto 1/2 MS plates or 1/2 MS medium supplemented with either 150 mM of NaCl or 250 mM of sorbitol, and cultivated for 11 days to observe their root growth or for 6 h to perform qRT-PCR.

Total RNA was isolated from seedling samples by using the Eastep^®^Super (Promega, Fitchburg, United States) according to the protocols. Approximately 2 μg of total RNA was reverse transcribed into first-strand cDNA by using the First-Strand cDNA Synthesis SuperMix (TransScript, Beijing China). The qRT-PCR was performed on the 7500 Fast Real-Time PCR System (Applied Biosystems, Foster City, CA, United States) which used a Power SYBR^®^Green PCR Master Mix (TransStart, Beijing China). The *Arabidopsis Actin2* served as an internal control. The stress-responsive genes selected for detecting their expression are listed in Supplementary Table S2. All primers used in this study are listed in Supplementary Table S3. At least three independent biological replicates were performed for the qRT-PCR analysis. Value changes of more than twofold, >2 or <0.5, were considered to, respectively, indicate the induction or repression of gene expression. The data analysis was carried out using the Data Processing System, and a two-way analysis of variance (ANOVA) followed by Tukey’s multiple range test were conducted to determine any significant differences among the group means (Tang and Zhang, 2013).

## Results

### Expression of the Ca^2+^ Binding Protein PV in *Arabidopsis*

**FIGURE 1:**
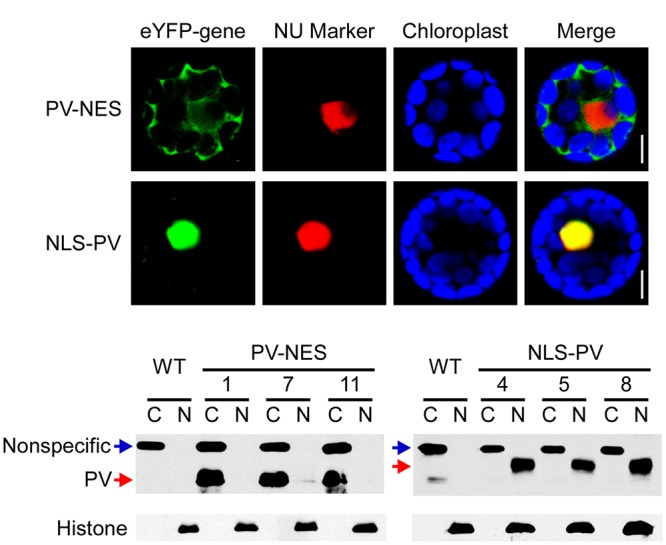
Subcellular localization of PV-NES and NLS-PV in the transgenic *Arabidopsis*. **(A)** Localization of eYFP-PV-NES and eYFP-NLS-PV after being transiently co-transformed with a mCherry-labeled nuclear marker in the *Arabidopsis* mesophyll cells. Green indicates eYFP, red indicates the nuclear marker *BES-mCherry*, and the blue indicates the chloroplast autofluorescence. All images in this figure were obtained from one optic section. Scale bars are equivalent to 10 μm. **(B)** Western blot to detect the subcellular localization of PV-NES and NLS-PV in the transgenic *Arabidopsis* plants. An equal amount of protein (10 μg) was loaded into each lane. Histone is used as the marker of the nucleus component, and non-specific band as the marker of the cytoplasm component.

To study the relationship between cytosolic and nucleosolic calcium signaling in plants, we constructed binary vectors targeting the Ca^2+^-binding protein PV to either the nucleus or cytoplasm, by fusing it with a NLS (NLS-PV) or a NES (PV-NES), and transducing the vectors into *Arabidopsis* WT via *Agrobacterium*. Meanwhile, to verify the localization signal, two vectors with the indicated PV fused with yellow fluorescent protein—eYFP-PV-NES and eYFP-NLS-PV (**Supplementary Figure S1**)—were generated and transiently co-transformed with the mCherry-labeled nuclear marker into the *Arabidopsis* mesophyll protoplasts. We found that eYFP-PV-NES was restricted to the cytoplasm, whereas eYFP-NLS-PV was co-localized with the nuclear marker (**Figure 1A**). Through resistance screening and the reverse transcription (RT)-PCR assay (data not shown), we firstly discarded the transgenic lines which has different growth phenotype with WT in whole life cycle, then obtained the single-insertion, T_3_ homozygous *NLS-PV* and *PV-NES* transgenic lines. So, the proteins extracted from the cytoplasm and nucleus of the three independent lines were isolated and detected by immunoblotting, revealing that the PV was distributed only in the nuclear compartment in the *NLS-PV* lines and in the cytoplasm compartment in the *PV-NES* lines (**Figure 1B**, the original images of **Figure 1B** is shown in (**Supplementary Figure S3**). These results proved that the subcellular localization of PV-NES or NLS-PV is specific to the cytoplasm or nucleus in the transgenic plants, which lay the foundation for its selective buffering of [Ca^2+^]_cyt_ or [Ca^2+^]_nuc_.

### PV-NES Attenuates the Osmotic Stress-Induced [Ca^2+^]_cyt_ Increase (OICI_cyt_) and Salt Stress-Induced [Ca^2+^]_cyt_ Increase (SICI_cyt_)

**FIGURE 2:**
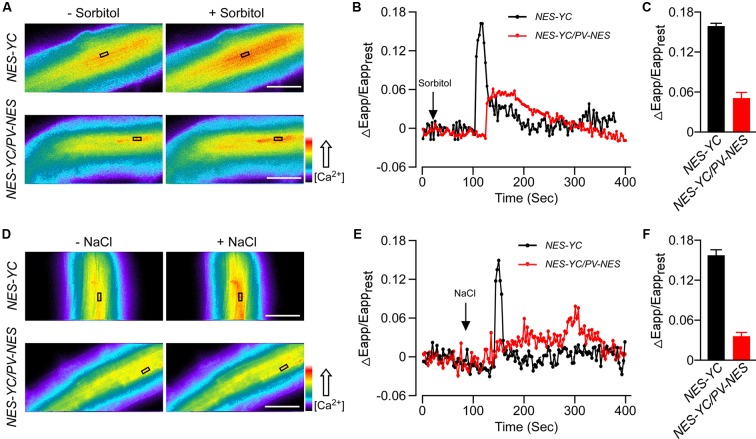
Osmotic stress-induced [Ca^2+^]_cyt_ increase (OICI_cyt_) and salt stress-induced [Ca^2+^]_cyt_ increase (SICI_cyt_) were impaired in the *PV-NES* transgenic lines. **(A,D)** OICI_cyt_ and SICI_cyt_ were detected in the *Arabidopsis* root cells expressing *NES-YC* and *NES-YC/PV-NES* before and after treatment with 250 mM of sorbitol **(A)** or 125 mM of NaCl **(D)**, respectively. Regions of interest used to monitor OICI_cyt_ and SICI_cyt_ in **(B,E)** are indicated by black rectangles. Scale bar in the image is equivalent to 50 μm. Relative [Ca^2+^]_cyt_ is shown as the emission fluorescence ratios (F535/F480) and scaled by a pseudo-color bar (bottom right). **(B,E)** Time courses of OICI_cyt_ and SICI_cyt_ in the *Arabidopsis* root cells expressing *NES-YC* (black curve) and *NES-YC/PV-NES* (red curve) treated with 250 mM of sorbitol **(B)** and 125 mM of NaCl **(E)**, respectively. The [Ca^2+^]_cyt_ is shown as the change in the apparent FRET efficiency. **(C,F)** Impairment effects of PV-NES on OICI_cyt_ **(C)** and SICI_cyt_ **(F)** in the root cells. Treatments are the same as described in **(B,E)** above (*n* = 20 to 30 cells from at least 9 different seedlings, each of which included 3 to 6 root cells).

To explore the effect of PV on the cellular [Ca^2+^] elevations, we crossed the *PV-NES* lines with those *Arabidopsis* plants containing the cytoplasm-localized Yellow Cameleon 3.6 Indicator (*NES-YC*), and thus obtained the homozygous *NES-YC/PV-NES* lines with resistance screening. Then, the [Ca^2+^]_cyt_ was monitored in response to different stimuli in the root cells of *Arabidopsis* plants either expressing *NES-YC* or co-expressing *NES-YC/PV-NES*. Firstly, we found that 250 mM of sorbitol, when used as an osmotic stress stimulus, can trigger a rapid increase of [Ca^2+^]_cyt_ in the root cells of the *NES-YC* plants; the value of ΔEapp/Eapp_rest_ reached 0.16, which is similar to that of a previous study (Krebs et al., 2012). However, OICI_cyt_ was disrupted in the *NES-YC/PV-NES* lines, with a ΔEapp/Eapp_rest_ value that was approximately 0.05 (**Figures 2A–C**). Similarly, 125 of mM NaCl was also able to induce a single peak of [Ca^2+^]_cyt_ increase in the root cells of the *NES-YC* plants, but it was impaired in the *NES-YC/PV-NES* lines (**Figures 2D–F**). The increased FRET fluorescence and decreased CFP fluorescence after adding the 250 mM of sorbitol or 125 mM of NaCl to the root cells of the *NES-YC* plants are shown in (**Supplementary Figures S2A,B**). Together, these results demonstrated that the cytosolic-localized PV can effectively buffer the change of [Ca^2+^]_cyt_ and thereby inhibit OICI_cyt_ and SICI_cyt_ in the *Arabidopsis* root cells.

### NLS-PV Impaired the Osmotic Stress-Induced [Ca^2+^]_nuc_ Increase (OICI_nuc_) and Salt Stress-Induced [Ca^2+^]_nuc_ Increase (SICI_nuc_)

**FIGURE 3:**
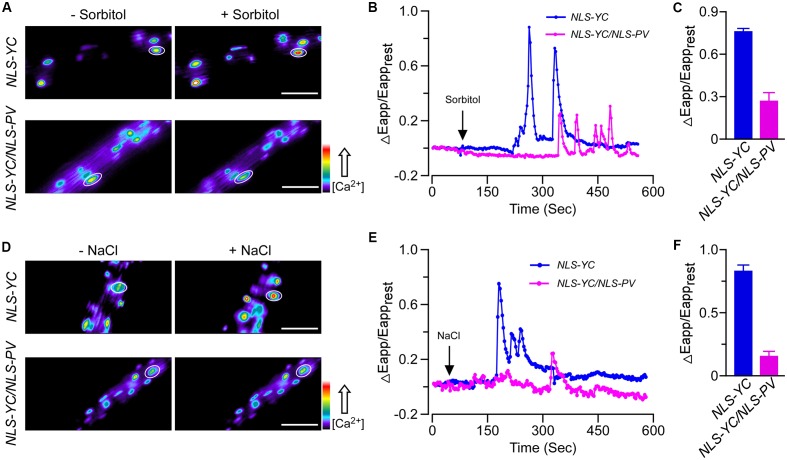
Osmotic stress-induced [Ca^2+^]_nuc_ increase (OICI_nuc_) and salt stress-induced [Ca^2+^]_nuc_ increase (SICI_nuc_) were disrupted in the *NLS-PV* transgenic lines. **(A,D)** OICI_nuc_ and SICI_nuc_ were detected in *Arabidopsis* root cells expressing *NLS-YC* and *NLS-YC/NLS-PV* before and after treatment with 250 mM of sorbitol **(A)** or 125 mM of NaCl **(D)**, respectively. Regions of interest used to monitor OICI_cyt_ and SICI_cyt_ in **(B,E)** are indicated by white ellipses. Scale bar in the image is equivalent to 50 μm. Relative [Ca^2+^]_cyt_ is shown as the emission fluorescence ratios (F535/F480) and scaled by a pseudo-color bar (bottom right). **(B,E)** Time courses of OICI_nuc_ and SICI_nuc_ in the *Arabidopsis* root cells expressing *NES-YC* (blue curve) and *NES-YC/PV-NES* (purple curve) after treatment with 250 mM of sorbitol **(B)** and 125 mM of NaCl **(E)**, respectively. The [Ca^2+^]_nuc_ is shown as the change in the apparent FRET efficiency. **(C,F)** Impairment effects of NLS-PV on OICI_nuc_ **(C)** and SICI_nuc_ **(F)** in the root cells. Treatments are the same as described in **(B,E)** above (*n* = 20 to 30 cells from at least 9 different seedlings, each of which included 3 to 6 root cells).

We also obtained the homozygous *NLS-YC/NLS-PV* lines and used these plants to monitor the calcium elevations in the nucleus after adding 250 mM sorbitol or 125 mM NaCl. Firstly, we found that OICI_nuc_ in the root cells of *NLS-YC* plants rapidly increased and oscillated, with a peak value for ΔEapp/Eapp_rest_ of approximately 0.82. However, OICI_nuc_ was disrupted in the *NLS-YC/NLS-PV* lines for which the value of ΔEapp/Eapp_rest_ was approximately 0.28 (**Figures 3A–C**). In addition, the calcium oscillation pattern of SICI_nuc_ was also impaired in the *NLS-YC/NLS-PV* lines compared with that of the *NLS-YC* plants, and the ΔEapp/Eapp_rest_ values had decreased from 0.8 to 0.2 (**Figures 3D–F**). (**Supplementary Figures S2C,D**) show the increased FRET fluorescence and decreased CFP fluorescence in the root cells of the *NLS-YC* plants after adding 250 mM sorbitol or 125 mM NaCl treatments. These results showed that nuclear-localized PV could also buffer OICI_nuc_ and SICI_nuc_ in *Arabidopsis*.

### Changes in [Ca^2+^]_cyt_ Triggered by Osmotic or Salt Stresses Are Independent of Those for [Ca^2+^]_nuc_ in *Arabidopsis*, and Vice Versa

**FIGURE 4:**
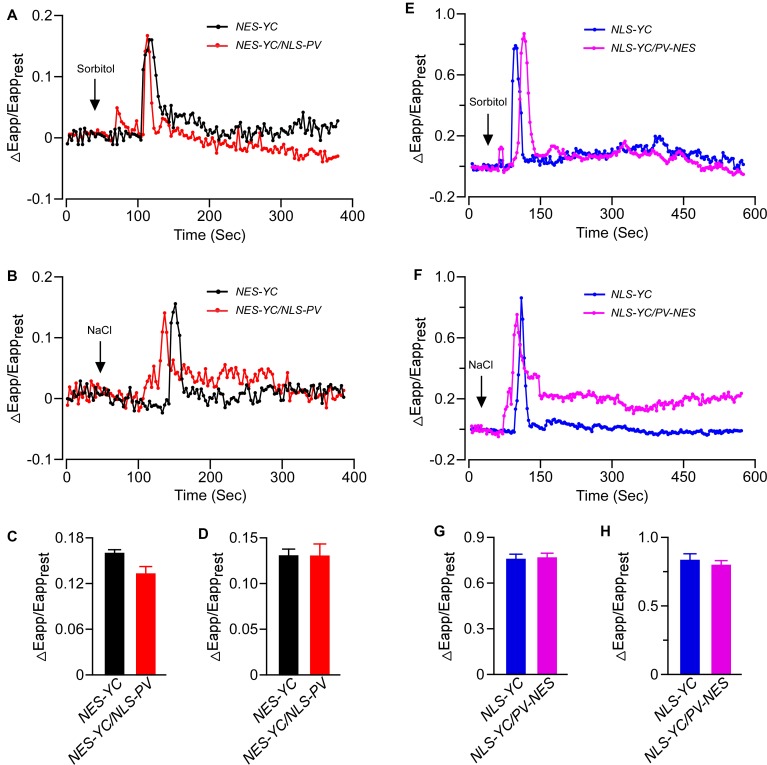
The increases of [Ca^2+^]_cyt_ and [Ca^2+^]_nuc_ triggered by osmotic stress or salt stress were independent of each other. **(A,B)** Time courses of OICI_cyt_ and SICI_cyt_ detected in the *Arabidopsis* root cells expressing *NES-YC* (black curve) and *NES-YC/NLS-PV* (red curve) after treatment with 250 mM of sorbitol **(A)** or 125 mM of NaCl **(B)**, respectively. The [Ca^2+^]_cyt_ is shown as the change in the apparent FRET efficiency. **(C,D)** Impairment effects of NLS-PV on OICI_cyt_ **(C)** and SICI_cyt_ **(D)** in the root cells. The values are means ± SD (*n* = 20 to 30 cells). **(E,F)** Time courses of OICI_nuc_ and SICI_nuc_ detected in the *Arabidopsis* root cells expressing *NLS-YC* (blue curve) and *NLS-YC/PV-NES* (purple curve) after treatment with 250 mM of sorbitol **(E)** or 125 mM of NaCl **(F)**, respectively. The [Ca^2+^]_cyt_ is shown as the change in the apparent FRET efficiency. **(G,H)** Impairment effects of PV-NES on OICI_nuc_ **(G)** and SICI_nuc_ **(H)** in the root cells. The values are means ± SD (*n* = 20 to 30 cells from at least 9 different seedlings, each of which included 3 to 6 root cells).

To further reveal the inter-relationship between [Ca^2+^]_cyt_ and [Ca^2+^]_nuc_, we intercrossed *NEC-YC* with *NLS-PV* and *NLS-YC* with *PV-NES* plants. Doing so gave us the homozygous *NEC-YC/NLS-PV* and *NLS-YC/PV-NES* double-transgenic lines for detecting the OICI and SICI in the cytoplasm and nucleus, respectively. Firstly, the responses, in terms of the pattern and ΔEapp/Eapp_rest_, for the cytoplasm calcium dynamics in the root cells of the *NEC-YC/NLS-PV* plants were similar to those seen in the *NES-YC* plants when they received 250 mM of sorbitol or 125 mM of NaCl (**Figures 4A–D**). This shows that blocking nuclear calcium has no effect on OICI_cyt_ and SICI_cyt_. Likewise, we separately measured the change of [Ca^2+^]_nuc_ in response to the 250-mM sorbitol or 125-mM NaCl treatments in the root cells of the *NLS-YC* and *NLS-YC/PV-NES* lines and found that blocking the cytosolic calcium with PV-NES did not affect OICI_nuc_ and SICI_nuc_ (**Figures 4E–H**). Interestingly, we want to show that the increase of [Ca^2+^]_nuc_ in response to osmotic or salt stresses showed two kinds of patterns in the different cells of the root mature zone: one is a rapid increase following by oscillations (**Figures 3B,E**), while the other is just a single peak (**Figures 4E,F**). Each pattern accounted for approximately 50%. In addition, blocking the cytosolic calcium signal with PV-NES had no effect on these two patterns of [Ca^2+^]_nuc_. Via the Western blot, we also confirmed that PV is expressed in all these intercrossed lines (**Supplementary Figure S4**). In order to rule out any bias about the increases of [Ca^2+^]_cyt_ and [Ca^2+^]_nuc_ due to stimulus application in this study, we perform the control experiment where only MS medium is added to the seedlings and show that the medium cannot trigger any change of ΔEapp/Eapp_rest_ (**Supplementary Figure S5**). These results proved that cytosolic and nucleosolic calcium dynamics are independent of each other in *Arabidopsis*.

### Both Cytosolic and Nucleosolic Calcium Are Involved in Root Growth and the Transcription of Several Abiotic Stress-Induced Genes in *Arabidopsis*

**FIGURE 5:**
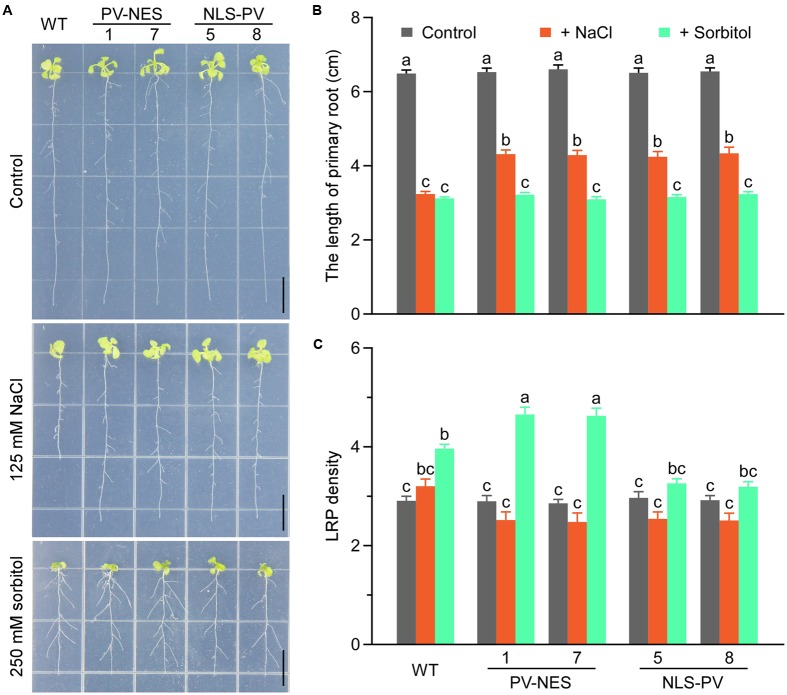
The seedling adaptation in response to the salt or osmotic stresses in *Arabidopsis*. **(A)** Effect of salt or osmotic stress in the WT, *NES-PV*, and *NLS-PV* transgenic lines. The 3-day-old seedlings were transplanted into plates of 125 mM NaCl or 250 mM sorbitol, and grown for 11 more days. Scale bars indicate 1 cm. **(B)** Primary root length of the WT, *NES-PV*, and *NLS-PV* plants, with statistical results taken from **(A)**; error bars are *SE*, *n* = 40 seedlings. **(C)** Lateral root primordium (LRP) density in **(A)**; error bars are *SE*, *n* = 40 seedlings. The “a,” “b,” “c” letters are used to denote significance among the means (*P* < 0.05, Student’s *t*-test). Bars sharing the same letter are not significantly different.

To better distinguish the respective roles of cytosolic and nucleosolic calcium signaling in plant growth and development, we detected the root growth of WT, *PV-NES*, and *NLS-PV* plants under conditions of abiotic stress. As expected, we showed that NLS-PV and PV-NES have no effects on normal root growth in the 1/2 MS medium, and that both the treatments of 125 mM NaCl and 250 mM sorbitol can inhibit the root growth of the WT, *PV-NES*, and *NLS-PV* plants. However, the root length of both the *PV-NES* and *NLS-PV* plants were markedly longer than that of the WT plants when the medium contained 125 mM of NaCl, but not 250 mM of sorbitol. This indicated that the cytosolic and nucleosolic calcium signaling participated in the response to salt stress regulating the root growth in *Arabidopsis* (**Figures 5A,B**). In addition, treatment with 250 mM sorbitol induced the development of lateral root primordia at a high density in the WT roots, while the lateral primordia density of *PV-NES* plants was greater than that of either the WT or *NLS-PV* plants. Nevertheless, the 125-mM NaCl treatment led to no obvious differences in the development of lateral primordia among the WT, *PV-NES*, and *NLS-PV* plants (**Figures 5A,C**). Together, these results suggested that cytosolic calcium, but not nucleosolic calcium, is involved in the osmotice stress response regulating the growth of lateral root primordia in *Arabidopsis*.

**FIGURE 6:**
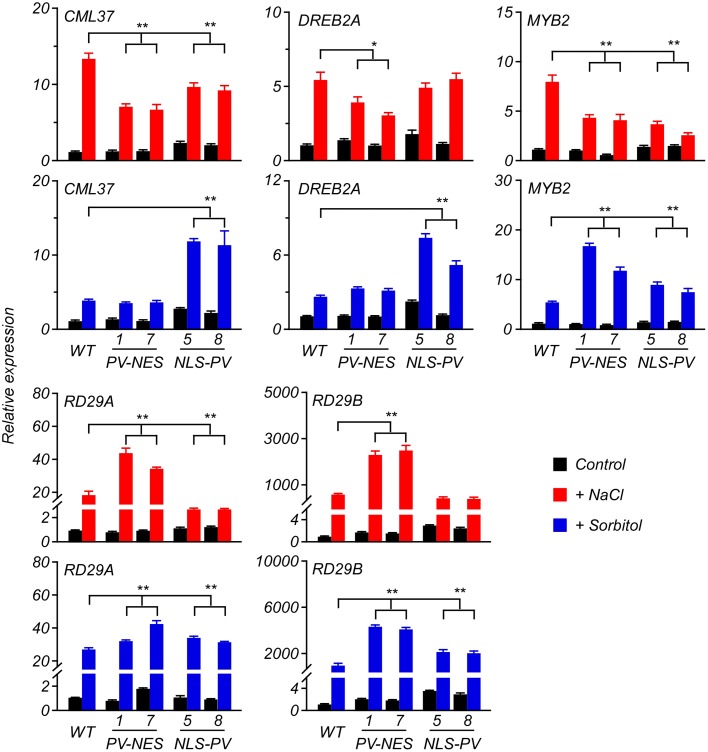
Expression of several genes in response to the salt or osmotic stresses in the 6-day-old *Arabidopsis* seedlings. The qRT-PCR analyses were performed to detect the transcript abundance of *CML37*, *DREB2A*, *MYB2*, *RD29A*, and *RD29B* responding to 125 mM of NaCl or 250 mM of sorbitol applied to the seedlings. Error bars are *SD*, *n* = 3 biological replicates. ^∗^*P* < 0.05, ^∗∗^*P* < 0.001 (two-way ANOVA followed by a Tukey’s multiple comparisons test), which represent the significant difference of gene expression in transgenic lines compared with WT after the stresses treatment.

Previous studies revealed that several genes, such as *CML37* (Scholz et al., 2015), *DREB2A* (Sakuma et al., 2006), *MYB2* (Abe, 2002), *RD29A* (Msanne et al., 2011), *RD29B* (Msanne et al., 2011; Virlouvet et al., 2014) and *RD22* (Harshavardhan et al., 2014), are stress-responsive genes in *Arabidopsis*; so, here we performed qRT-PCR to detect whether their expression was related to cytosolic and/or nucleosolic calcium. Compared with the expression of the genes up-regulated by the sorbitol or NaCl treatment in WT, we found that the up-regulated level of *CML37* and *MYB2* were inhibited in both *PV-NES* and *NLS-PV* plants, whereas that of *DREB2A* was inhibited only in the *PV-NES* plants and that of *RD29A* only in *NLS-PV* plants after receiving the 125-mM NaCl treatment. However, the up-regulated levels of *RD29A* and *RD29B* were higher in the *PV-NES* than in WT plants after treatment with 125 mM of NaCl. More interestingly, the sorbitol treatment-induced expression of *CML37* and *DREB2A* was higher in the *NLS-PV* lines than for the WT and *PV-NES* plants, and the transcription of *MYB2*, *RD29A*, and *RD29B* were greater in both *PV-NES* and *NLS-PV* lines than those in WT (**Figure 6**). We also found the expression pattern of *RD22* after treatment with osmotic or salt stresses stayed the same in the *PV-NES* and *NLS-PV* plants as in WT (**Supplementary Figure S6**). These results suggested that [Ca^2+^]_cyt_ and [Ca^2+^]_nuc_ both participate in the various transcription of stress-related plant genes.

## Discussion

Drought and salt stress are major abiotic constraints that are capable of impairing plant growth and development and inflicting severe crop losses worldwide (Munns and Tester, 2008). The first phase common to both drought and salt stress is characterized by osmotic stress (Shavrukov, 2013). Here, we demonstrated that osmotic and salt stresses induced [Ca^2+^]_nuc_ and [Ca^2+^]_cyt_ increases in *Arabidopsis* root cells. These results suggested that both cytosolic and nucleosolic calcium serve as the secondary messengers involved with plant responses to various environmental stresses and developmental processes. In agreement with studies of animal cells (Pusl et al., 2002; Rodrigues et al., 2007), we further proved that when PV is targeted to the cytoplasm or nucleoplasm it could buffer OICI_cyt_ and SICI_cyt_, or OICI_nuc_ and SICI_nuc_, respectively. This approach could be established in plants for the first time to study how Ca^2+^ operates and functions for different stimuli in different plant tissues/organs.

Using the organelle-targeted Ca^2+^ indicator, Cameleon YC3.6, to produce stably transformed *Arabidopsis* plants lets us monitor organelle-specific Ca^2+^ dynamics and compare the [Ca^2+^] kinetics between different organelles in *planta* (Krebs et al., 2012; Loro et al., 2012; Stael et al., 2012; Bonza et al., 2013). For example, Loro et al. (2012) showed that mitochondrial Ca^2+^ accumulation is strictly related to the intensity of the cytosolic Ca^2+^ increase, demonstrating a tight association between mitochondrial and cytosolic Ca^2+^ dynamics. Generally, the endoplasmic reticulum (ER) acts as a Ca^2+^ store that releases Ca^2+^ for stimulus-induced [Ca^2+^]_cyt_ increases; however, Bonza et al. (2013) showed that Ca^2+^ elevations in ER are followed by various stimuli-induced [Ca^2+^]_cyt_ increases in *Arabidopsis* root-tip cells, with distinct dynamics, which does not support a significant role of ER [Ca^2+^] as a source of Ca^2+^ release that contributes to the formation of cytosolic Ca^2+^ signatures. In this study, we investigated the triggering of OICI_cyt_ by 250 mM of sorbitol and that of SICI_cyt_ by 125 mM of NaCl in the root cells of the *NES-YC* transgenic lines. We found a similar pattern of OICI_cyt_ and SICI_cyt_, featuring a single peak of [Ca^2+^] elevation—consistent with findings for the root cells of aequorin-transgenic *Arabidopsis* seedlings (Kiegle et al., 2000; Tracy et al., 2008)—and a sustained and peak-decreased OICI_cyt_ and SICI_cyt_ pattern in the *NEC-YC/PV-NES* root cells (**Figures 2B,E**). More interestingly, OICI_nuc_ and SICI_nuc_ in the root cells of the *NLS-YC* lines showed two distinct patterns: the transient increases and oscillation of [Ca^2+^], with each pattern about approximately 50% in the different cells of the mature root zone (**Figures 3B,E**, **4E,F**). In addition, we found that the targeted PV in the nucleus can block OICI_nuc_ and SICI_nuc_ in the root cells of *NLS-YC/NLS-PV* lines. However, Loro et al. (2012) showed that osmotic stress-induced Ca^2+^ transients in the nucleoplasm are kinetically similar to those in the cytoplasm of *Arabidopsis* guard cells. These results indicated that the temporal and spatial characteristics of calcium signatures present in different plant organs in response to various stimuli.

Extensive studies show that the same stimuli—such as osmotic shocks triggered by high concentration of sorbitol or NaCl (Mithöfer and Mazars, 2002), jasmonic acid (JA) and its biosynthetic precursor OPDA (Walter et al., 2007), ATP or Nod factor (Krebs et al., 2012)—may trigger increases of both [Ca^2+^]_nuc_ and [Ca^2+^]_cyt_ yet this has diverse patterns in different plants. In addition, the outer nuclear membrane is bordered by the endoplasmic reticulum, so they share a common Ca^2+^ pool, Ca^2+^-permeable channels, and Ca^2+^-ATPase carriers to produce Ca^2+^ signaling in plants. In this study, we first used the PV-NES fusion protein to buffer the [Ca^2+^]_cyt_ increases to demonstrate a stimulus-induced [Ca^2+^]_nuc_ that was independent of [Ca^2+^]_cyt_ with distinct dynamics. Similarly, blocking [Ca^2+^]_nuc_ with NLS-PV has no effect on the stimulus-induced [Ca^2+^]_cyt_ increases in the *Arabidopsis* root cells. These results show that cytosolic and nucleosolic calcium signaling are independent of each other in *Arabidopsis*, which raises two interesting questions: (i) how do plants sense and transduce extracellular stimuli to simultaneously induce the [Ca^2+^] increases in the cytoplasm and nucleoplasm, and (ii) what is the physiological extent and function of organelle-specific calcium signaling?

Osmotic and salt stress inhibition of plant growth and development is a general phenomenon, and a pressing and interesting scientific issue. Here, we revealed that *PV-NES* and *NLS-PV* plants exhibit a reduced salt stress-mediated inhibition of root growth, but not of osmotic stress-mediated inhibition of root growth; this suggests that salt stress-induced [Ca^2+^] increases in both the cytoplasm and nucleoplasm mediate the salt stress-induced growth inhibition in *Arabidopsis*. Another interesting result we found is the lateral root primordia density of *PV-NES* plants exceeding those of WT and *NLS-PV* plants, but only when treated with 250 mM of sorbitol; this indicates that the OICI_cyt_ is somehow involved in the osmotic-stress mediated development of lateral roots. Prior studies have foundthat calcium signaling is crucial for plant adaptation to various stresses and that it participates in rapid changes in gene expression (Dodd et al., 2010; Reddy et al., 2011).

Compared with the up-regulated expression of key stress-responsive genes, *CML37* (Scholz et al., 2015), *DREB2A* (Sakuma et al., 2006), *MYB2* (Abe, 2002), *RD29A* (Msanne et al., 2011), *RD29B* (Msanne et al., 2011; Virlouvet et al., 2014) in the WT, they were expressed differently in the *PV-NES* and *NLS-PV* plants after the NaCl and sorbitol treatments, respectively (**Figure 6**). These results further implied that cytosolic calcium signaling and nuclear calcium signaling function independently in the stress response pathways of plants. We also found that the expression of some genes, for instance *RD22*, is related neither to cytosolic or nucleosolic calcium in response to osmotic or salt stresses in *Arabidopsis*. This indicates that one or more calcium-independent signaling pathway(s) participated in the expression regulation of *RD22* in *Arabidopsis* plants. It is also possible that overexpression of PV-NES or NLS-PV themselves could affect the gene expression in a [Ca^2+^]-independent pathway in the transgenic *Arabidopsis* plants. Further experiments are needed to address this issue. For example, we can generate transgenic plants overexpressing Ca^2+^-binding deficient mutants of PV (Pusl et al., 2002), and demonstrate that these PV mutants cause changes in gene expression in a calcium-independent manner. Recently, high-throughput sequencing approaches have become available, capable of generating large expression data profiles which provides a useful tool for characterizing the stress-responsive gene(s) mediated by different calcium signaling pathways using the *PV-NES* and *NLS-PV* plants. In sum, our study is the first to show that cytosolic and nucleosolic calcium dynamics are mutually independent in plants, yet play coexisting roles critical in regulating gene expression for plant adaptation to various environmental stresses.

## Author Contributions

Conceived and designed the experiments: SH, FH, and JL. Performed the experiments: FH, JL, TN, WC, and XJ. Analyzed the data: FH, JL, HZ, YW, and SH. Contributed reagents/materials/analysis tools: YW and SH. Wrote the paper: FH, HZ, YW, and SH.

## Conflict of Interest Statement

The authors declare that the research was conducted in the absence of any commercial or financial relationships that could be construed as a potential conflict of interest.

## Footnotes

1 http://www.arabidopsis.org/

## Supplementary Material

FIGURE S1 Schematics of the different PV constructs. Fusion proteins were constructed, consisting of PV with NES fused to the C-terminal region (i.e., PV-NES) and NLS to the N-terminal region (i.e., NLS-PV), and eYFP fused to the N-terminal of these two fusion proteins (i.e., eYFP-PV-NES and eYFP-NLS-PV). The 35S promoter sequence is indicated by the light-blue arrow, the 3XHA tag by the purple rectangular box, the PV by the yellow rectangular box, the NLS by the deep blue rectangular box, the NES by the green rectangular box, the eYFP by the light-green rectangular box, the NOS terminator by the red arrow, Hygromycin is the plant selection marker (denoted by the gray rectangle box), and the T-DNA border is depicted as an empty rectangle box. The line bar is the length of 200 base pairs (bp).

FIGURE S2 The changes in CFP and CpVenus intensities that were used to calculate the apparent FRET efficiency. **(A)** 250-mM sorbitol treatment of the *NES-YC* transgenic lines shown in **Figure 2B**. **(B)** 125-mM NaCl treatment of the *NES-YC* transgenic lines shown in **Figure 2D**. **(C)** 250-mM sorbitol treatment of the *NLS-YC* transgenic lines shown in **Figure 3B**. **(D)** 125-mM NaCl treatment of the *NLS-YC* transgenic lines shown in **Figure 3D**.

FIGURE S3 The original image of **Figure 1B**: Western blot to detect the subcellular localization of PV-NES and NLS-PV in the transgenic *Arabidopsis* plants. An equal amount of protein (10 μg) was loaded into each lane. Histone is used as the marker of the nucleus component, and non-specific band as the marker of the cytoplasm component.

FIGURE S4 Western blot to detect the PV levels in the WT and different transgenic plants. An equal amount of protein (10 μg) was loaded into each lane. The non-specific band is used as the marker of the loading control.

FIGURE S5 The change of [Ca^2+^]_cyt_ or [Ca^2+^]_nuc_ response to bathing medium as the stimuli in transgenic plants roots. **(A)** *NES-YC* transgenic lines. **(B)** *NES-YC/PV-NES* transgenic lines. **(C)** *NES-YC/NLS-PV* transgenic lines. **(D)** *NLS-YC* transgenic lines. **(E)** *NLS-YC/NLS-PV* transgenic lines. **(F)** *NLS-YC/NES-PV* transgenic lines. Each measurement was examined at about six individual seedling roots, which include 3∼6 cells for every root.

FIGURE S6 The transcription level of RD22 in response to the treatment with a high concentration of sorbitol and NaCl in 6-day-old seedlings of the WT, *NES-PV*, and *NLS-PV* plants, as detected via qRT-PCR. Error bars are *SD*, *n* = 3 biological replicates. ^∗^*P* < 0.05, ^∗∗^*P* < 0.001 (two-way ANOVA followed by Tukey’s multiple comparisons test).

## Abbreviations

CAS-IP_3_: Ca^2++^-sensing receptor-inositol trisphosphate
CCaMK: Ca^2+^/calmodulin-dependent Ser/Thr protein kinase
CFP: cyan fluorescent protein
CNGC15: cyclic nucleotide-gated channel 15
DMI1: does not Make Infections 1
DTT: dithiothreitol
EGF: epidermal growth factor
eYFP: enhanced yellow fluorescent protein
GLRs: glutamate receptor-like channels
LRP: lateral root primordium
MKK: mitogen-activated protein kinase kinase
NES: nuclear export signal
NLS: nuclear localized signal
OICI_cyt_: osmotic stress-induced cytosolic [Ca^2+^] increase
OICI_nuc_: osmotic stress-induced nucleosolic [Ca^2+^] increase
OPDA: 12-oxophytodienoic acid
PV: parvalbumin
PVDF: polyvinylidene fluoride
SDS: sodium dodecyl sulfate
SERCA: sarco/endoplasmic reticulum Ca^2+^-ATPase
SICI_cyt_: salt stress-induced cytosolic [Ca^2+^] increase
SICI_nuc_: salt stress-induced nucleosolic [Ca^2+^] increase
WT: wild type
YC: Cameleon YC 3.6.
