# Supplementary figures and images for: Cytosolic and Nucleosolic Calcium Signaling in Response to Osmotic and Salt Stresses Are Independent of Each Other in Roots of Arabidopsis Seedlings
Source: Front Plant Sci. 2017 Sep 21;8:1648. doi: 10.3389/fpls.2017.01648 (PMC5613247; doi:10.3389/fpls.2017.01648)

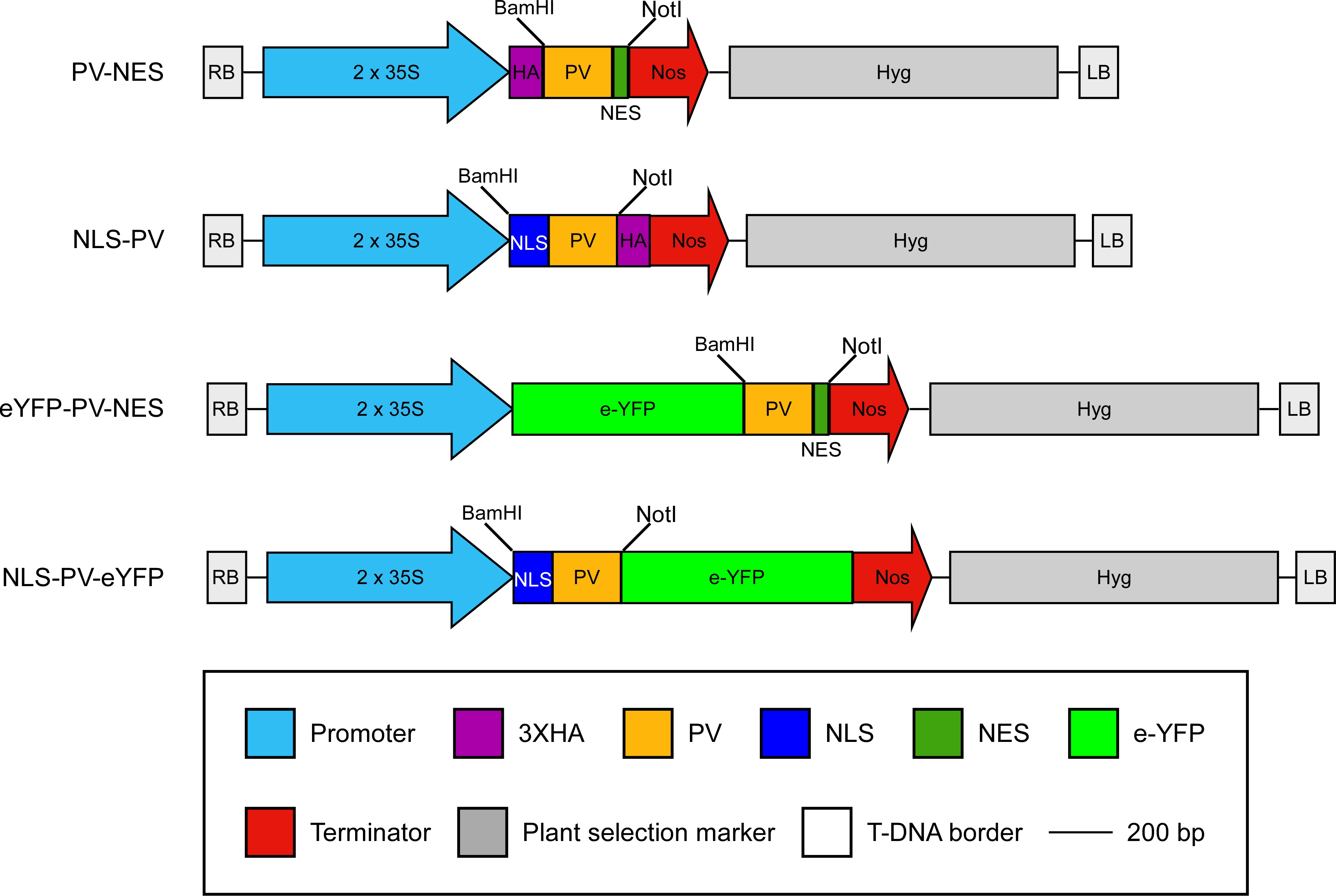

Supplement: FIGURE S1 — Schematics of the different PV constructs. Fusion proteins were constructed, consisting of PV with NES fused to the C-terminal region (i.e., PV-NES) and NLS to the N-terminal region (i.e., NLS-PV), and eYFP fused to the N-terminal of these two fusion proteins (i.e., eYFP-PV-NES and eYFP-NLS-PV). The 35S promoter sequence is indicated by the light-blue arrow, the 3XHA tag by the purple rectangular box, the PV by the yellow rectangular box, the NLS by the deep blue rectangular box, the NES by the green rectangular box, the eYFP by the light-green rectangular box, the NOS terminator by the red arrow, Hygromycin is the plant selection marker (denoted by the gray rectangle box), and the T-DNA border is depicted as an empty rectangle box. The line bar is the length of 200 base pairs (bp). [file Image_1.JPEG]

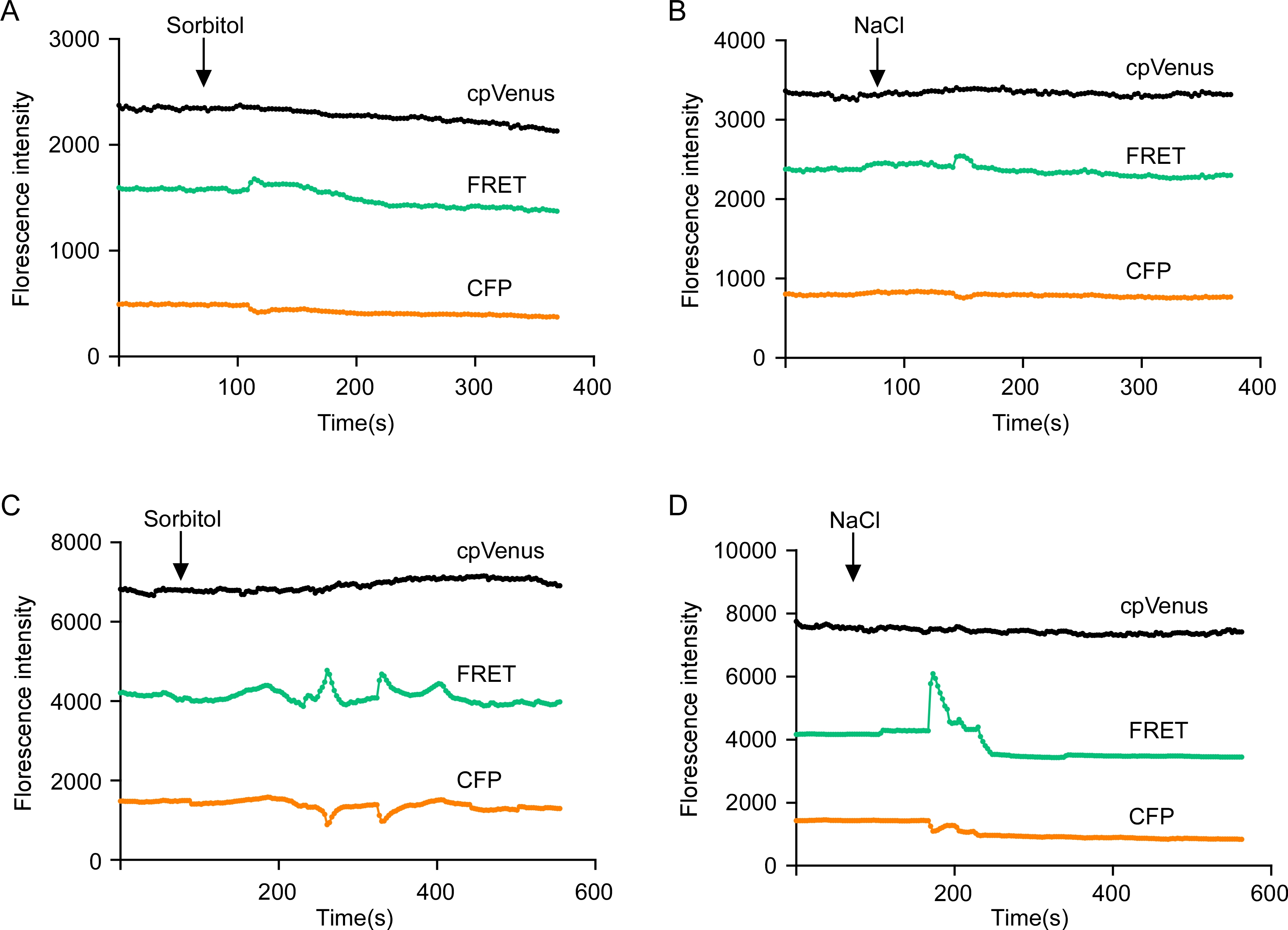

Supplement: FIGURE S2 — The changes in CFP and CpVenus intensities that were used to calculate the apparent FRET efficiency. (A) 250-mM sorbitol treatment of the NES-YC transgenic lines shown in Figure 2B. (B) 125-mM NaCl treatment of the NES-YC transgenic lines shown in Figure 2D. (C) 250-mM sorbitol treatment of the NLS-YC transgenic lines shown in Figure 3B. (D) 125-mM NaCl treatment of the NLS-YC transgenic lines shown in Figure 3D. [file Image_2.JPEG]

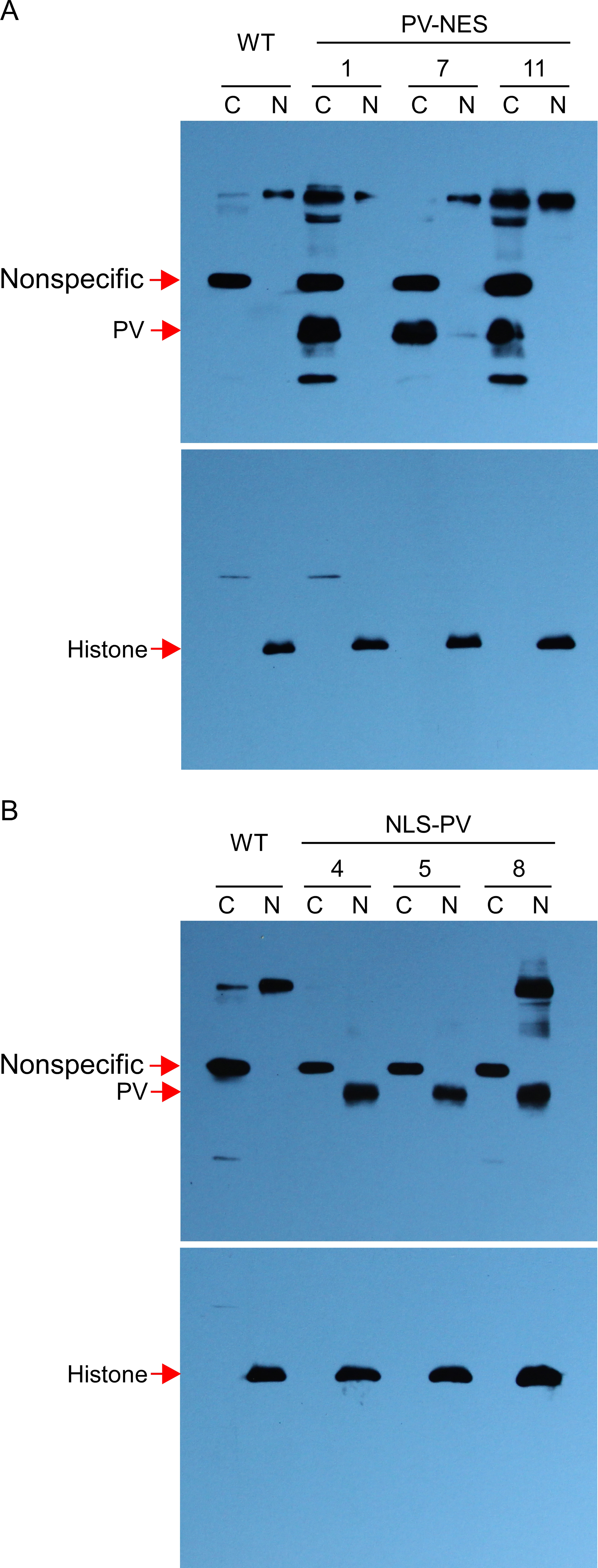

Supplement: FIGURE S3 — The original image of Figure 1B: Western blot to detect the subcellular localization of PV-NES and NLS-PV in the transgenic Arabidopsis plants. An equal amount of protein (10 μg) was loaded into each lane. Histone is used as the marker of the nucleus component, and non-specific band as the marker of the cytoplasm component. [file Image_3.JPEG]

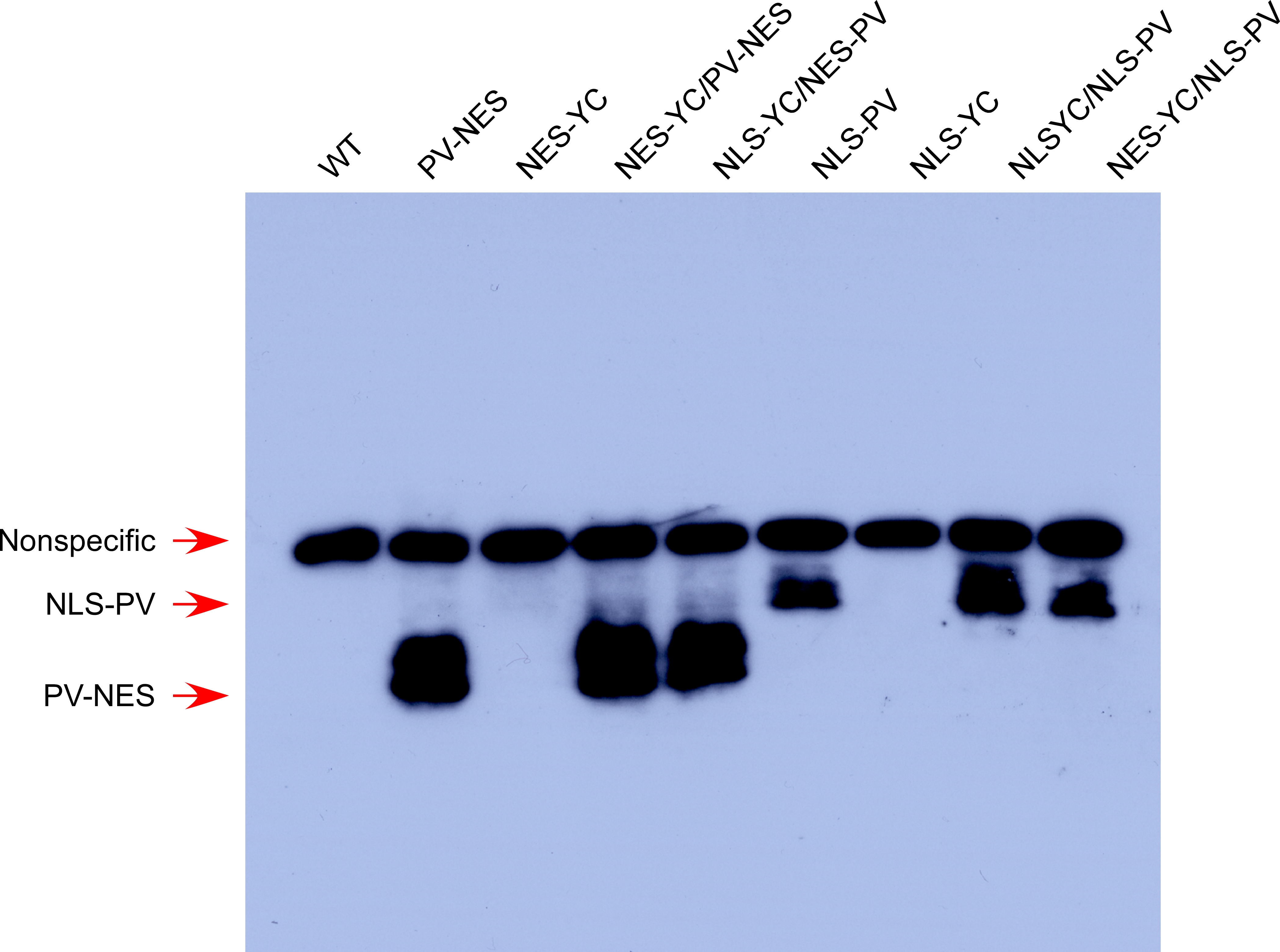

Supplement: FIGURE S4 — Western blot to detect the PV levels in the WT and different transgenic plants. An equal amount of protein (10 μg) was loaded into each lane. The non-specific band is used as the marker of the loading control. [file Image_4.JPEG]

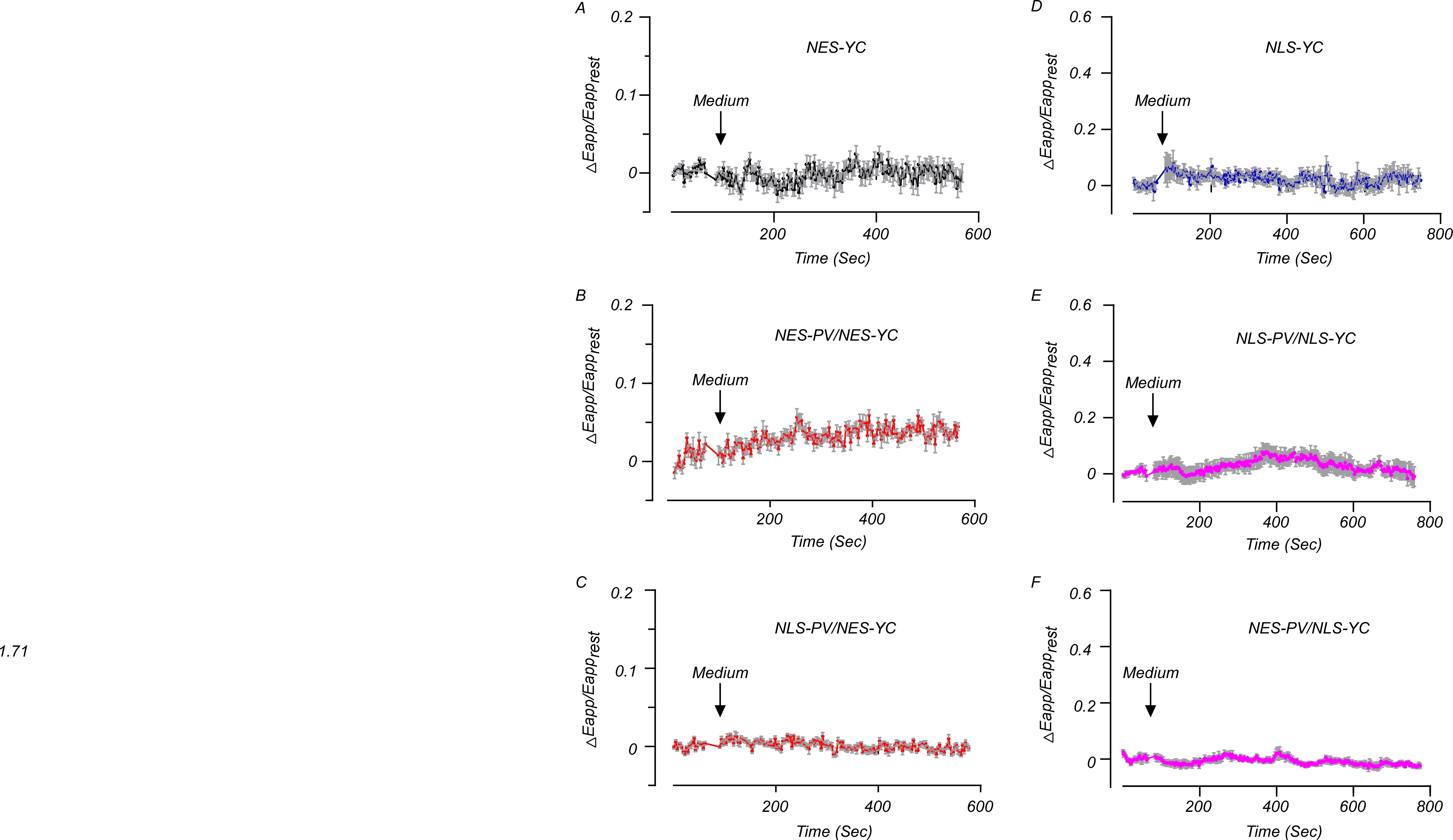

Supplement: FIGURE S5 — The change of [Ca2+]cyt or [Ca2+]nuc response to bathing medium as the stimuli in transgenic plants roots. (A) NES-YC transgenic lines. (B) NES-YC/PV-NES transgenic lines. (C) NES-YC/NLS-PV transgenic lines. (D) NLS-YC transgenic lines. (E) NLS-YC/NLS-PV transgenic lines. (F) NLS-YC/NES-PV transgenic lines. Each measurement was examined at about six individual seedling roots, which include 3∼6 cells for every root. [file Image_5.JPEG]

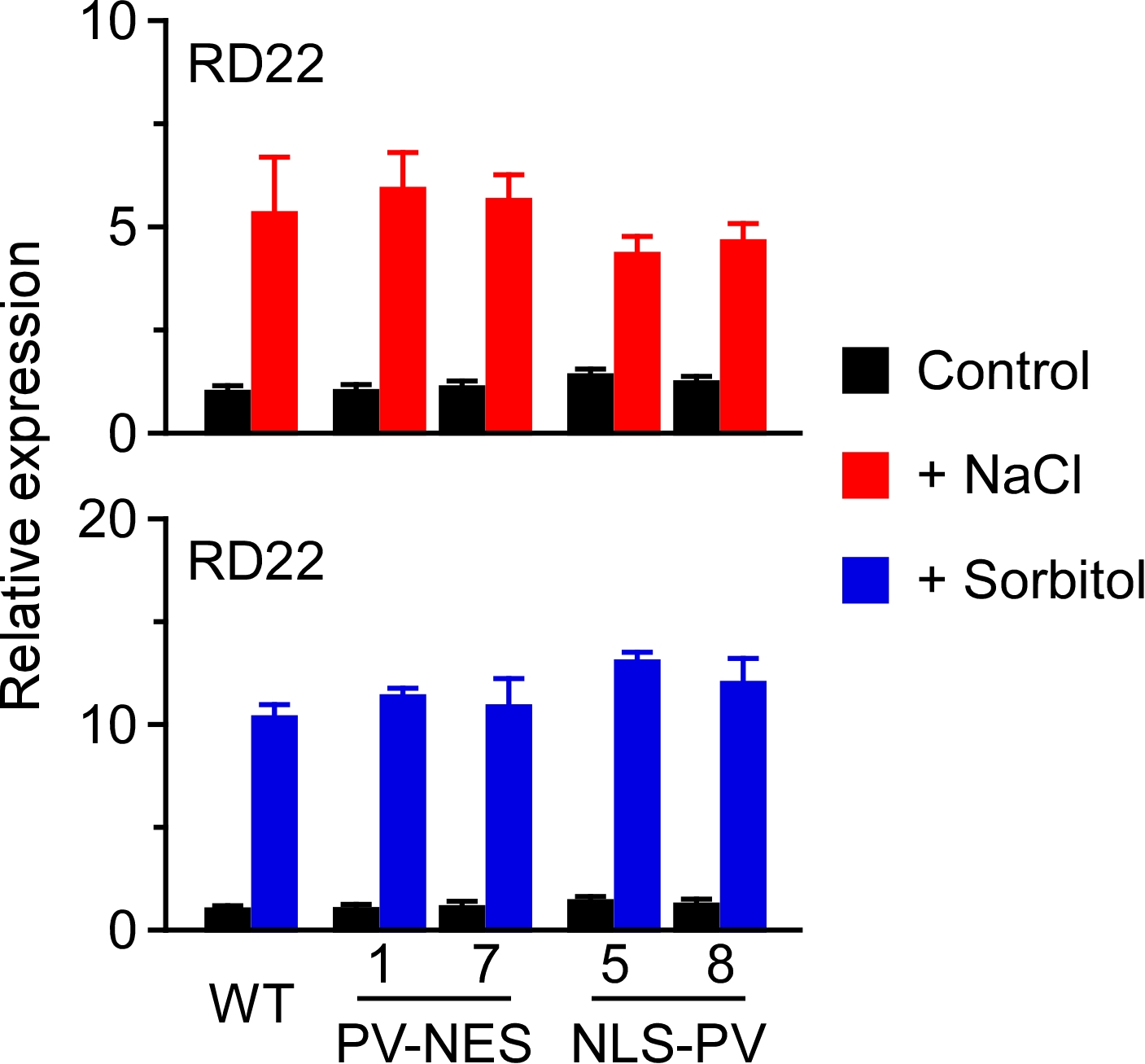

Supplement: FIGURE S6 — The transcription level of RD22 in response to the treatment with a high concentration of sorbitol and NaCl in 6-day-old seedlings of the WT, NES-PV, and NLS-PV plants, as detected via qRT-PCR. Error bars are SD, n = 3 biological replicates. ∗P < 0.05, ∗∗P < 0.001 (two-way ANOVA followed by Tukey’s multiple comparisons test). [file Image_6.JPEG]
