# Supplementary material for: Cytosolic and Nucleosolic Calcium Signaling in Response to Osmotic and Salt Stresses Are Independent of Each Other in Roots of Arabidopsis Seedlings
Source: Front Plant Sci. 2017 Sep 21;8:1648. doi: 10.3389/fpls.2017.01648 (PMC5613247; doi:10.3389/fpls.2017.01648)
Supplement: Supplementary file 7 [file Table_1.PDF]

Table S1: Primer sequences used for cloning *PV*, *NES fragment* and *NLS fragment*, and constructing the plant expression vectors.

Primer	Sequence (5'-3')	Length
NLS-*Bam* HI-FOR	CGGGATCCATGACGTCTGACGGAGCAACGT	30
NLS-*Hind* III-REV	CCCAAGCTTGGGTCTTCTCCGCCGCTC	27
PV-*Hind* III-FOR	CCCAAGCTTATGTCGATGACAGACTTGCTCA	34
PV-*Not* I-REV	ATAAGAATGCGGCCGCGCTTTCGGCCACCAGAG	33
PV-NES-*Bam* HI-FOR	CGGGATCCATGTCGATGACAGACTTGCTCA	36
PV-NES-*Not* I-REV	ATAAGAATGCGGCCGCTCAAAGCTTTTCATCTAATT<br>CTAATTCTTCTAATTTTTTTTGTAATGCCATGCTTTC<br>GGCCAC	79
