# Supplementary material for: Cytosolic and Nucleosolic Calcium Signaling in Response to Osmotic and Salt Stresses Are Independent of Each Other in Roots of Arabidopsis Seedlings
Source: Front Plant Sci. 2017 Sep 21;8:1648. doi: 10.3389/fpls.2017.01648 (PMC5613247; doi:10.3389/fpls.2017.01648)
Supplement: Supplementary file 8 [file Table_2.PDF]

Table S2. The marker genes responsible to osmotic- or salt-stress for qRT-PCR analysis in this study.

Gene	Stress/signal/role
CML37	JA, ABA, drought (Scholz et al., 2015)
DREB2A	Drought, cold (Sakuma et al., 2006)
MYB2	Salt, ABA (Abe, 2002)
RD29A, RD29B, RD22	Cold, drought, ABA (Msanne et al., 2011; Virlouvet et al., 2014)

Abe, H. (2002). Arabidopsis AtMYC2 (bHLH) and AtMYB2 (MYB) function as transcriptional activators in abscisic acid signaling. *Plant Cell* 15(1), 63-78. doi: 10.1105/tpc.006130.

Msanne, J., Lin, J., Stone, J.M., and Awada, T. (2011). Characterization of abiotic stress-responsive Arabidopsis thaliana RD29A and RD29B genes and evaluation of transgenes. *Planta* 234(1), 97-107. doi: 10.1007/s00425-011-1387-y.

Sakuma, Y., Maruyama, K., Osakabe, Y., Qin, F., Seki, M., Shinozaki, K., et al. (2006). Functional analysis of an Arabidopsis transcription factor, DREB2A, involved in drought-responsive gene expression. *Plant Cell* 18(5), 1292-1309. doi: 10.1105/tpc.105.035881.

Scholz, S.S., Reichelt, M., Vadassery, J., and Mithofer, A. (2015). Calmodulin-like protein CML37 is a positive regulator of ABA during drought stress in Arabidopsis. *Plant Signal Behav* 10(6), e1011951. doi: 10.1080/15592324.2015.1011951.

Virlouvet, L., Ding, Y., Fujii, H., Avramova, Z., and Fromm, M. (2014). ABA signaling is necessary but not sufficient for RD29B transcriptional memory during successive dehydration stresses in Arabidopsis thaliana. *Plant J* 79(1), 150-161. doi: 10.1111/tpj.12548.
