# Supplementary material for: Cytosolic and Nucleosolic Calcium Signaling in Response to Osmotic and Salt Stresses Are Independent of Each Other in Roots of Arabidopsis Seedlings
Source: Front Plant Sci. 2017 Sep 21;8:1648. doi: 10.3389/fpls.2017.01648 (PMC5613247; doi:10.3389/fpls.2017.01648)
Supplement: Supplementary file 9 [file Table_3.PDF]

Table S3. Primers sequence for qRT-PCR

Primer	Sequence (5'-3')	Length
CLM37-F	TGGATGCAAACTCAGACGGGA	21
CLM37-R	ACCACTTCCTCCACCTCACG	22
DREB2A-F	CGAGGGAAAGGATGGTAATGG	27
DREB2A-R	CGTTGTGGGATTAAGGCAAATATC	31
MYB2-R	ATTGAACGAAACCCGGCTCG	20
MYB2-F	GTGGAACGAATCAACGCCCA	20
RD29A-F	TTCTGGCGAAGGGAAGACCT	20
RD29A-R	TGGGTCTCTTCCCAGCTCAG	20
RD29B-F	ACAGCTTTGGAAAATGGAGTCACAG	25
RD29B-R	CTCATGATGCTCTTCTTCTTCTGGA	25
RD22-F	ACGATCCTAACGCGGCTCTC	20
RD22-R	AAGGCACCGTTTCAGCCTCT	20
